# Commentary: Is preclinical diabetic retinopathy in diabetic nephropathy individuals more severe?

**DOI:** 10.3389/fendo.2023.1256555

**Published:** 2023-09-22

**Authors:** Yusuke Kameda

**Affiliations:** Yotsuya-sanchome Ekimae Eye Clinic, Tokyo, Japan

**Keywords:** diabetic retinopathy, diabetic nephropathy, retinal microvascular impairment, estimated glomerular filtration rate (eGFR), case-control study

## 1 Introduction

We read the recent publication by Yao and Li [Yao H, Li Z. Is preclinical diabetic retinopathy in diabetic nephropathy individuals more severe?. *Front. Endocrinol*. (2023) 14:1144257] with considerable interest (1). The authors concluded that preclinical diabetic retinopathy (DR) may be more severe in individuals with diabetic nephropathy (DN) than in non-diabetic nephropathy (NDN) individuals with regard to microvascular and microstructural impairment. Moreover, estimated glomerular filtration rate (eGFR) may be a good indicator of retinal microvascular impairment (1). In addition to reading the study by Yao and Li, we have been investigating the association between diabetic eye disease (including the diagnostic or therapeutic agents for the disease) and DN for many years and have published several related papers (2–7). We support and appreciate the authors’ work and agree with their conclusions but have some concerns about their methods and results. Therefore, we would like to provide critical comments on these issues.

## 2 Is this a matched case–control study?

In the patient demographics summarized in Table 1 of the article, the two groups (NDN and DN groups) had exactly the same numbers of cases and male-to-female ratios (NDN versus DN: case: 44 versus 44; male-to-female ratio: 30:16 versus 30:16). Age and HbAlc levels were also fairly consistent between the two groups (age: 58.86 ± 11.60 versus 59.80 ± 12.55; HbAlc levels: 8.96 ± 2.88 versus 8.91 ± 1.89). The study design seemed to be an age-, sex-, and HbAlc level-matched pair case–control study (exact matching, with a ratio of 1:1). However, in the Methods section, the authors wrote that the study design was a retrospective case–control study. Moreover, the authors did not state anywhere in the article that controls included age, sex, and HbAlc levels matched in a ratio of 1:1. Therefore, there was insufficient disclosure of information about the statistical methods. If this was a matched pair case–control study, the authors should have described the correct study design, as in the study by Karat et al. (8).

## 3 Is the number of patients correct?

The authors stated that this study included 88 eyes of 88 patients (44 NDN and 44 DN). However, the numbers of male and female patients in Table 1 were 46 in each group; this exceeds the number of cases reported. Similarly, the number of diabetic mellitus therapy regimen of the DN group in Table 1 also appears to be discordant. We are concerned that similar miscalculations may have occurred in the data for other major outcomes. Therefore, we recommend that the authors reexamine the data of their study to determine whether the continuous variables were over- or underestimated.

## 4 Discussion

Data from our previous studies indicate that a decline in eGFR could have a detrimental impact on DR, and the study by Yao and Li confirmed our hypothesis. However, several of the issues that we have pointed out have compromised the quality of their manuscript. Therefore, we hope the authors provide responses to these issues that satisfy the readers.

## Author contributions

YK: Writing – original draft, Writing – review & editing.

## References

[1] Yao H Li Z. Is preclinical diabetic retinopathy in diabetic nephropathy individuals more severe? Front Endocrinol (2023) 14:1144257. doi: 10.3389/fendo.2023.1144257 PMC10064084 37008921

[2] Kameda Y Babazono T Haruyama K Iwamoto Y Kitano S. Renal function following fluorescein angiography in diabetic patients with chronic kidney disease. Diabetes Care (2009) 32:e31. doi: 10.2337/dc08-1692 19246582

[3] Kameda Y Hirose A Iida T Uchigata Y Kitano S. Giant retinal pigment epithelial tear associated with fluid overload due to end-stage diabetic kidney disease. Am J Ophthalmol Case Rep (2017) 5:44–7. doi: 10.1016/j.ajoc.2016.11.004 PMC5758016 29503946

[4] Kameda Y Babazono T Uchigata Y Kitano S. Renal function after intravitreal administration of vascular endothelial growth factor inhibitors in patients with diabetes and chronic kidney disease. J Diabetes Investig (2018) 9:937–9. doi: 10.1111/jdi.12771 PMC6031523 29108104

[5] Kameda Y Hanai K Uchigata Y Babazono T Kitano S. Vitreous hemorrhage in diabetes patients with proliferative diabetic retinopathy undergoing hemodialysis. J Diabetes Investig (2020) 11:688–92. doi: 10.1111/jdi.13161 PMC7232265 31618517

[6] Kameda Y Saeki T Hanai K Suzuki Y Uchigata Y Babazono T. Is chronic kidney disease affecting the postoperative complications of vitrectomy for proliferative diabetic retinopathy? J Clin Med (2021) 10:5309. doi: 10.3390/jcm10225309 34830589 PMC8621452

[7] Kameda Y Suzuki Y Sugai M Ishinabe K Fukuoka N. Comment on: Long-term hemodialysis improved and stabilized diabetic macular edema: two case reports. Int J Ophthalmol (2022) 15:1891–2. doi: 10.18240/ijo.2022.11.25 PMC9631177 36404974

[8] Karat S Lobo AC Satish D Devaraj R Manjooran RR Nithyanandam S. Uncontrolled diabetes mellitus exacerbated by COVID-19-induced inflammation is the risk factor for COVID-19-associated rhino-orbito-cerebral mucormycosis: A matched pair case-control study. Indian J Ophthalmol (2022) 70:3096–101. doi: 10.4103/ijo.IJO_448_22 PMC9672762 35918980

